# Meta-analysis and review of rechargeable implantable pulse generators for spinal cord stimulation and deep brain stimulation

**DOI:** 10.1016/j.bas.2024.104148

**Published:** 2024-11-26

**Authors:** Denizhan Özgan, Sandro M. Krieg, Martin Jakobs

**Affiliations:** a Medical School of İstanbul Okan University, İstanbul, Turkiye; b Department of Neurosurgery, Heidelberg University Hospital, Heidelberg, Germany; c Division of Stereotactic Neurosurgery, Department of Neurosurgery, Heidelberg University Hospital, Heidelberg, Germany; d Heidelberg University, Medical Faculty, Heidelberg, Germany

**Keywords:** Rechargeable, Implantable pulse generator, Deep brain stimulation, Spinal cord stimulation

## Abstract

**Introduction:**

Neuromodulation through deep brain stimulation (DBS) and spinal cord stimulation (SCS) has become a successful therapy for various neurological disorders, such as movement disorders and chronic pain. Implantable pulse generators (IPGs) are pivotal in these therapies, available as either rechargeable (r-IPGs) or non-rechargeable (nr-IPGs).

**Research question:**

To perform a meta-analysis on r-IPGs.

**Methods:**

A systematic literature search following PRISMA guidelines was conducted on PubMed, focusing on studies published from January 2005 to August 2023. Included studies comprised clinical trials, randomized controlled trials, and comparative studies involving human subjects. Data extraction focused on patient demographics, stimulation types, battery characteristics, and complications. Descriptive statistics and Pearson correlation analyses were performed using SPSS software.

**Results:**

Nine studies involving 288 patients with rechargeable IPGs (r-IPGs) for SCS and 257 patients with r-IPGs for DBS met the inclusion criteria. r-IPGs exhibited low rates of surgical revisions and infections, with surgical revision rates of 8.87% for SCS and 5.45% for DBS, and infection rates of 2.6% for SCS and 1.56% for DBS. Charge burden was comparable with 97.34 min and 93.41 min per week for SCS and DBS respectively. Correlation analyses indicated that longer battery recharge times were associated with an increased incidence of complications, including unintentional interruptions and hardware failures.

**Discussion:**

r-IPGs may offer substantial benefits in reducing re-operation rates and complications associated. Nonetheless, careful management of battery charging is crucial to maximize these benefits. Establishing international guidelines for the use of r-IPGs in specific patient populations and conditions is recommended to standardize and optimize outcomes.

## Highlights

• Longer battery charging times are associated with interruptions of therapy for SCS
Longer intervals between recharges are associated with recharging problems for SCS
• r-IPGs are associated with low rates of complications and especially infections
• Interruptions of therapy due to problems with recharging affected every 10th patient
• The external parts of the r-IPG system fail more often than the implanted parts.

## Abbreviations and acronyms:

DBS: deep brain stimulation
nr-IPG: non-rechargeable implantable pulse generator
OCD: obsessive compulsive disorder
PD: Parkinson's Disease
r-IPG: rechargeable implantable pulse generator
SCS: spinal cord stimulation

## 1 Introduction

Various movement disorders such as Parkinson's Disease (PD) (Brown et al., 1999; Deuschl et al., 2006; Schuepbach et al., 2013), tremor (Benabid et al., 1991; Pahwa et al., 1999), and dystonia (Kupsch et al., 2011), along with refractory epilepsy (Fisher et al., 2010) and obsessive-compulsive disorder (OCD) (Lakhan and Callaway, 2010), have been successfully treated using deep brain stimulation (DBS), a method that has been clinically established in the 1990s. Furthermore, spinal cord stimulation (SCS) (Hajiabadi et al., 2023) has proven to be an effective approach for addressing refractory chronic and neuropathic pain in the trunk of the body and its extremities (Shealy et al., 1967).

The fundamental power source for these neurostimulation interventions, which deliver regular electrical impulses to the electrodes, are implantable pulse generators (IPGs). These generators exist in two categories: rechargeable and non-rechargeable (Jakobs et al., 2019). The initially developed non-rechargeable IPGs (nr-IPGs) possess a fixed battery capacity and require replacement when their lifespan concludes (S et al., 1967; Hornberger et al., 2008). The lifespan of batteries depends on the fundamental parameters of stimulation and the battery model (Israeli-Korn et al., 2019). On average, these batteries have a lifespan ranging from 2 to 5 years, making frequent replacements unavoidable (Hornberger et al., 2008; Costandi et al., 2020).

The depletion of battery life is the primary cause of re-operation among DBS patients (Boviatsis et al., 2010; Latini et al., 2015) Although battery replacement surgeries are less extensive than the initial DBS implantation surgery, they can entail significant complications due to specific inherent risks (Thrane et al., 2014). The potential for treatment discontinuation due to infections that can follow these replacement surgeries is not a situation to be taken lightly (Helmers et al., 2018a). Furthermore, it has been demonstrated that the probability of this occurrence notably rises following the third battery replacement in DBS patients (Fytagoridis et al., 2016; Helmers et al., 2018b).

Rechargeable implantable pulse generators (r-IPGs), which have been employed for SCS since 2004 and for DBS since 2008, introduce a much distinct perspective (Ahmadi et al., 2021). These batteries, with their smaller size and an expected lifespan of up to 25 years, are becoming increasingly prominent (J et al., 2019; Hitti et al., 2018). Despite being usually more expensive than nr-IPGs, the overall cost-effectiveness is evident when considering the expenses of revision surgeries and the costs for new replacement implants (Willems et al., 2023). Furthermore, due to its prolonged lifespan, it not only eliminates the need for further surgeries but also mitigates the inherent risks in any procedure, such as the possibility of infection. This emphasizes that r-IPGs offer a highly appealing alternative (Taylor et al., 2020). However, it is crucial for patients to consistently monitor their battery charge levels and adhere to charging procedures, as a mistake during charging or fully depleting the battery can potentially result in its malfunction. The aim of this article is to assess and compare the incidence of medical interventions (such as battery replacement, infections, battery depletion, etc.) necessitated by r-IPGs.

## 2 Methods

### 2.1 Systematic literature search

A systematic literature search was conducted following the guidelines of Preferred Items for Reporting Systematic Reviews and Meta-analyses (Page et al., 2021) (PRISMA). A search of the electronic database PubMed was performed to identify the existing literature investigating the effects of r-IPGs to neurostimulation surgeries. The search terms included “rechargeable IPG”, “rechargeable neurostimulator”, “rechargeable pulse generator”, “and “rechargeable stimulator”. Literature search was narrowed to all available articles published from January 1st, 2005, to August 15th, 2023. To be included, studies were required to meet the following conditions: (a) clinical trial, randomized controlled trial and comparative study of r- IPGs; (b) written in English; (c) human subjects. Studies were excluded if (a) mention of complications and/or charging time, or (b) clinical data of the patients could not be identified.

### 2.2 Study quality assessment

The quality of each study was assessed using the classification scheme developed by French and Gronseth (2008). This scheme includes 4 levels of evidence, with level 1 representing high quality studies with low risk of bias and level 4 representing studies with a very high risk of bias.

### 2.3 Study selection

**Fig. 1:**
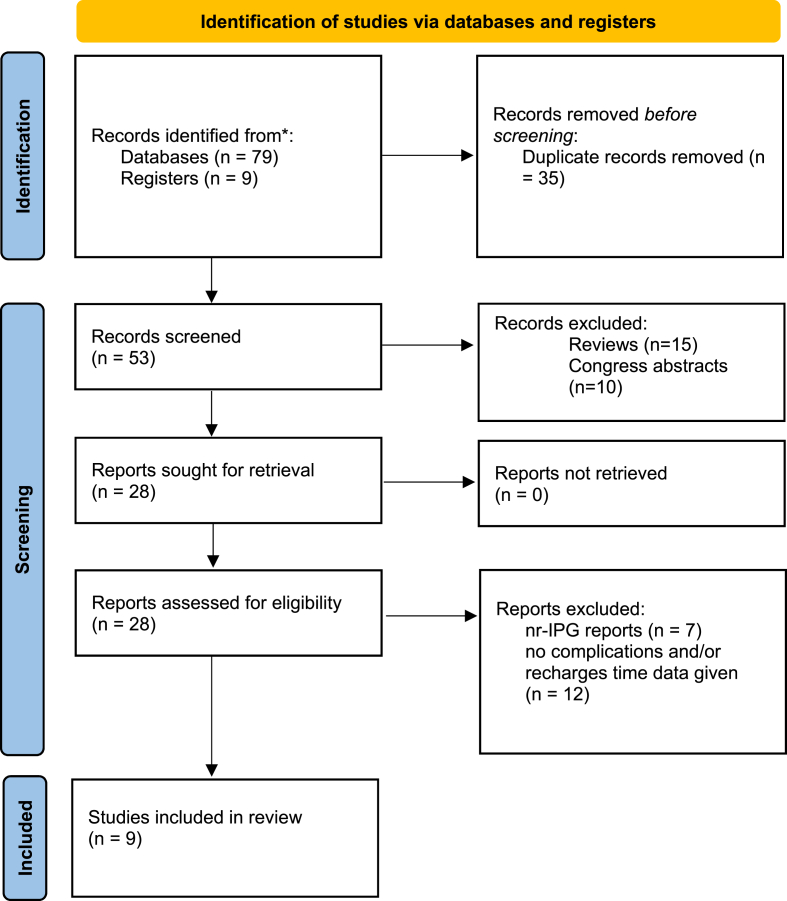
Adapted PRISMA 2020 flow diagram.

The PubMed search of the existing literature on the clinical outcome of r-IPG identified articles. Abstracts were screened for the above-mentioned selection inclusion criteria, which resulted in the exclusion 35 duplicate articles. After these articles were eliminated, articles were screened and n = 15 reviews and n = 10 congress abstracts were further removed. Full texts of the remaining 28 articles were subsequently checked for eligibility. N = 19 studies focusing on nr-IPGs and not containing relevant data were excluded from the analysis. In total, 9 studies were included as seen in Fig. 1 Of which 6 studies were controlled trials with an evidence level of three (French and Gronseth, 2008). 3 reports were randomized, double-blinded controlled trials, with an evidence level of two.

### 2.4 Data extraction

The following data were extracted from all studies included in the quantitative synthesis: first author name and publication year, number of participants, gender, operation type (DBS or SCS), IPG type, diagnosis, age at surgery. When possible, individual patient data was gathered from the constituent studies. The extracted data were shown in Table 1.

**Table 1:** Extracted Data (Overview of Included Studies n = 5 for SCS and n = 4 for DBS).

Stimulation Types	Studies	Number Of Patients	Battery Recharge Time (min)	Interval between charges (days)	COMPLICATIONS AND % VALUES							
					Infections	Surgical Revisions	Hard-Ware Failure of Implanted Parts	Hard-Ware Failure of External Stuff	Interruption Because of Recharging	Interruption Unintentionally	Skin Perforation	Unsuccessful Trials of Recharging
(SCS)	Hajiabadi et al. (Hajiabadi et al., 2023) (level 3)	40	113	10.24			0	16	11	11		0
		% Complications					0	40	27.5	27.5		0
	Van Buyten et al. (Van Buyten et al., 2008) (level 2)	41	108	15	1	11	3		1	0	0	1
		% Complications			2.44	26.83	7.31		2.44	0	0	2.44
	Kapural et al. (Kapural et al., 2015) (level 2)	171	45	1		8	8					0
		% Complications				.58	.58					0
	Kriek et al. (Kriek et al., 2017) (level 2)	28			1	3	3	0		1	0	0
		% Complications			3.57	10.71	10.71	0		3.57	0	0
	Reverberi et al. (Reverberi et al., 2013) (level 3)	8			0	0	0	0	0	0	0	0
		% Complications			0	0	0	0	0	0	0	0
Weighted Means for the Number of 5 SCS Studies		57.6	66.05	4.75	2.6	8.87	4.86	21.05	13.48	12.25	0	.34
(DBS)	Jakobs et al. (Jakobs et al., 2023) (level 3)	21	286	6.5	0	4	1	0	7	8	0	0
		% Complications			0	19.04	4.76	0	33.33	38.09	0	0
	Jakobs et al. (Jakobs et al., 2018) (level 3)	31	57.6	10.6	0	0	0	0	1	6	0	3
		% Complications			0	0	0	0	3.22	19.35	0	9.67
	Jakobs et al. (J et al., 2019) (level 3)	195	122	10	4	8	1	23	17	7	0	7
		% Complications			2.05	4.10	.51	11.79	8.72	3.59	0	3.59
	De Vloo et al. (De Vloo et al., 2018) (level 3)	10	93	2.2	0	2	1	9	2		1	0
		% Complications			0	20	10	90	20		10	0
Weighted Means for the Number of 4 DBS Studies		64.25	126.50	9.48	1.56	5.45	1.17	12.45	10.51	8.50	.39	3.89

### 2.5 Statistical analysis of data

The data analysed in this study are shown in tables and the results obtained by analysing the data with statistical methods are evaluated (SPSS Inc. Released, 2009. PASW Statistics for Windows, Version 18.0. Chicago: SPSS Inc). Among the data used in the analysis, the types of stimulation into two (2) groups and the other parameters were taken as they are. Stimulation Types: 1: SCS, 2: DBS. IPG Types: 1: r-IPG, 2: nr-IPG.

• Stimulation Types: 1: SCS, 2: DBS.
• Battery charging time is given as minutes.
• Interval between charges is given in number of days.
• Other parameters are taken as numerical values.

### 2.6 Descriptive statistics

Descriptive statistics were calculated for both SCS and DBS stimulation types, including the number of patients, battery recharge time, interval between charges, and various complications such as infection rates, surgical revisions, hardware failures, interruptions due to recharging problems, unintentional interruptions, skin perforation, and unsuccessful trials of recharging. These statistics provided insights into the distribution and characteristics of patients and outcomes across the included studies. Means, Std. Error, Std. Deviation and the values of Variance were calculated based on the number of patients. Descriptive statistics values were determined for the obtained data according to the stimulation types (SCS and DBS) and given in Table 2 and Table 3.

**Table 2:** Descriptive Statistics for SCS Stimulation Type (Complications are given as %).

	Range	Min.	Max.	Sum	Mean		Std. Deviation	Variance
	Statistic	Statistic	Statistic	Statistic	Statistic	Std. Error	Statistic	Statistic
Number Of Patients	163.00	8.00	171.00	288.00	57.6	25.91	57.93	6681.35
Battery Recharge Time (min)	68.00	45.00	113.00	266.00	66.05	22.13	38.33	675.00
Interval between charges (days)	14.00	1.00	15.00	26.24	4.75	4.07	7.05	.33
Infections	3.57	.00	3.57	6.01	2.6	1.15	1.99	2.59
Surgical Revisions	26.83	.00	26.83	38.12	8.87	2.72	5.44	21.92
Hardware Failures of Implanted Parts	10.71	.00	10.71	18.60	4.86	1.60	3.58	19.32
Hardware Failures of External Parts	40.00	.00	40.00	40.00	21.05	10.06	17.43	147.70
Interruption Be- cause of Rechar- ging Problems	27.50	.00	27.50	29.94	13.48	6.18	10.70	.00
Interruption Unin- tentionally	27.50	.00	27.50	31.07	12.25	5.17	10.35	37.77
Skin Perforation	.00	.00	.00	.00	.0	.00	.00	.00
Unsuccessful Trials of Recharging	2.44	.00	2.44	2.44	.34	.19	.42	.15

**Table 3:** Descriptive Statistics for DBS Stimulation Type (Complications are given as %).

	Range	Min.	Max.	Sum	Mean		Std. Devi- ation	Variance
	Statistic	Statistic	Statistic	Statistic	Statistic	Std. Error	Statistic	Statistic
Number Of Patients	185.00	10.00	195.00	257.00	64.25	37.93	75.85	10,803.52
Battery Recharge Time (min)	228.40	57.60	286.00	558.60	126.5	44.25	88.50	10,713.25
Interval between charges (days)	8.40	2.20	10.60	29.30	9.48	1.99	3.98	76.77
Infections	2.05	.00	2.05	2.05	1.56	.91	1.82	4.61
Surgical Revisions	20.00	.00	20.00	43.14	5.45	1.77	3.54	24.43
Hardware Failures of Implanted Parts	10.00	.00	10.00	15.27	1.17	.30	.60	.84
Hardware Failures of External Parts	90.00	.00	90.00	101.79	12.45	5.20	10.41	191.25
Interruption Be- cause of Rechar- ging Problems	30.11	3.22	33.33	65.27	10.51	3.69	7.37	101.12
Interruption Unin- tentionally	34.50	3.59	38.09	61.03	8.50	2.25	4.50	39.31
Skin Perforation	10.00	.00	10.00	10.00	.39	.23	.46	.29
Unsuccessful Trials of Recharging	9.67	.00	9.67	13.26	3.89	1.60	3.19	18.28

### 2.7 Correlation analysis

A Pearson Correlation analysis was performed to assess whether two of the data points have a strong positive or negative relationship with each other. Pearson Correlation Analysis was performed to ascertain the existence and strength of a relationship between the data for SCS and DBS stimulation type. The results are presented in Table 4, Table 5.

**Table 4:** Pearson correlation results for SCS stimulation type.

		Number Of Pa- tients	Battery Recharge Time (min)	Interval between charges (days)	Infections	Surgical Revisions	Hardware Fail- ures of Implanted	Hardware Fail- ures of External	Interruption Be- cause of Rechar-	Interruption Unin- tentionally	Skin Perforation	Unsuccessful Trials of Recharging
Number Of Patients	Correla- tion	1	−.999^a^	−.939	.924	.490	.907^a^	.778	.540	.469	.b	−.144
	Sig.		.034	.224	.250	.510	.033	.433	.637	.531	.	.817
	N	5	3	3	3	4	5	3	3	4	3	5
Battery Re- charge Time (min)	Correla- tion	−.999^a^	1	.919	.b	1.000^aa^	−.949	.b	1.000^aa^	1.000^b^	.b	.446
	Sig.	.034		.258	.	.	.204	.	.	.	.	.705
	N	3	3	3	1	2	3	1	2	2	1	3
Interval between charges (days)	Correla- tion	−.939	.919	1	.b	1.000^aa^	−.749	.b	1.000^aa^	−1.000^b^	.b	.763
	Sig.	.224	.258		.	.	.461	.	.	.	.	.448
	N	3	3	3	1	2	3	1	2	2	1	3
Infections	Correla- tion	.924	.b	.b	1	.712	1.000^aa^	.b	1.000^aa^	.500	.b	.500
	Sig.	.250	.	.		.496	.000	.	.	.667	.	.667
	N	3	1	1	3	3	3	2	2	3	3	3
Surgical Revi- sions	Correla- tion	.490	1.000^b^	1.000^aa^	.712	1	.590	.b	1.000^aa^	−.252	.b	.744
	Sig.	.510	.	.	.496		.410	.	.	.838	.	.256
	N	4	2	2	3	4	4	2	2	3	3	4
Hardware Failures of Implanted Parts	Correla- tion	.907^a^	−.949	−.749	1.00^b^	.590	1	−.500	−.431	−.540	.b	.035
	Sig.	.033	.204	.461	.000	.410		.667	.716	.460	.	.956
	N	5	3	3	3	4	5	3	3	4	3	5
Hardware Failures of External Parts	Correla- tion	.778	.b	.b	.b	.b	−.500	1	1.00^b^	.997	.b	.b
	Sig.	.433	.	.	.	.	.667		.	.052	.	.000
	N	3	1	1	2	2	3	3	2	3	2	3
Interruption Because of Recharging Problems	Correla- tion	.540	1.000^b^	−1.00^b^	1.00^b^	1.00^b^	−.431	1.000^b^	1	.997∗	.b	−.431
	Sig.	.637	.	.	.	.	.716	.		.050	.	.716
	N	3	2	2	2	2	3	2	3	3	2	3
Interruption Unintentionally	Correla- tion	.469	1.000^b^	1.00^b^	.500	−.252	−.540	.997	.997^a^	1	.b	−.373
	Sig.	.531	.	.	.667	.838	.460	.052	.050		.	.627
	N	4	2	2	3	3	4	3	3	4	3	4
Skin Perforation	Correla- tion	.b	.b	.b	.b	.b	.b	.b	.b	.b	.b	.b
	Sig.	.	.	.	.	.	.	.	.	.		.
	N	3	1	1	3	3	3	2	2	3	3	3
Unsuccessful Trials of Re- charging	Correla- tion	−.144	.446	.763	.500	.744	.035	.b	−.431	−.373	.b	1
	Sig.	.817	.705	.448	.667	.256	.956	.000	.716	.627	.	
	N	5	3	3	3	4	5	3	3	4	3	5

b. Cannot be computed because at least one of the variables is constant.

a Correlation is significant at the .05 level (2-tailed).

b Correlation is significant at the .01 level (2-tailed).

**Table 5:** Pearson correlation results for DBS stimulation type.

		Number Of Pa- tients	Battery Recharge Time (min)	Interval between charges (days)	Infections	Surgical Revisions	Hardware Failures of Implanted Parts	Hardware Failures of External Parts	Interruption Be- cause of Rechar-	Interruption Unin- tentionally	Skin Perforation	Unsuccessful Trials of
Number Of Patients	Correla- tion	1	−.131	.540	.994^bb^	.863	.382	.896^b^	.927^b^	.480	−.301	.941^b^
	Sig.		.869	.460	.001	.060	.526	.040	.023	.413	.622	.017
	N	5	4	4	5	5	5	5	5	5	5	5
Battery Re- charge Time (min)	Correla- tion	−.131	1	−.177	−.121	.330	.540	−.254	.243	.504	−.305	−.37 2
	Sig.	.869		.823	.879	.670	.460	.746	.757	.496	.695	.628
	N	4	4	4	4	4	4	4	4	4	4	4
Interval between charges (days)	Correla- tion	.540	−.177	1	.457	.190	−.571	.121	.382	.760	−.881	.740
	Sig.	.460	.823		.543	.810	.429	.879	.618	.240	.119	.260
	N	4	4	4	4	4	4	4	4	4	4	4
Infections	Correla- tion	.994^bb^	−.121	.457	1	.870	.408	.923^b^	.923^b^	.398	−.250	.907^b^
	Sig.	.001	.879	.543		.055	.495	.025	.025	.507	.685	.033
	N	5	4	4	5	5	5	5	5	5	5	5
Surgical Revi- sions	Correla- tion	.863	.330	.190	.870	1	.764	.837	.983^a^	.552	−.131	.680
	Sig.	.060	.670	.810	.055		.133	.077	.003	.334	.834	.206
	N	5	4	4	5	5	5	5	5	5	5	5
Hardware Failures of Im- planted Parts	Correla- tion	.382	.540	−.571	.408	.764	1	.580	.636	.277	.408	.149
	Sig.	.526	.460	.429	.495	.133		.306	.248	.652	.495	.811
	N	5	4	4	5	5	5	5	5	5	5	5
Hardware Failures of Ex- ternal Parts	Correla- tion	.896^b^	−.254	.121	.923^b^	.837	.580	1	.835	.167	.142	.783
	Sig.	.040	.746	.879	.025	.077	.306		.078	.789	.819	.117
	N	5	4	4	5	5	5	5	5	5	5	5
Interruption Be- cause of Re- charging Prob- lems	Correla- tion	.927^b^	.243	.382	.923^b^	.983^bb^	.636	.835	1	.616	−.273	.787
	Sig.	.023	.757	.618	.025	.003	.248	.078		.269	.657	.115
	N	5	4	4	5	5	5	5	5	5	5	5
Interruption Unintentionally	Correla- tion	.480	.504	.760	.398	.552	.277	.167	.616	1	−.603	.518
	Sig.	.413	.496	.240	.507	.334	.652	.789	.269		.282	.371
	N	5	4	4	5	5	5	5	5	5	5	5
Skin Perfora- tion	Correla- tion	−.301	−.305	−.881	−.250	−.131	.408	.142	−.273	−.603	1	−.363
	Sig.	.622	.695	.119	.685	.834	.495	.819	.657	.282		.549
	N	5	4	4	5	5	5	5	5	5	5	5
Unsuccessful Trials of Re- charging	Correla- tion	.941^b^	−.372	.740	.907^b^	.680	.149	.783	.787	.518	−.363	1
	Sig.	.017	.628	.260	.033	.206	.811	.117	.115	.371	.549	
	N	5	4	4	5	5	5	5	5	5	5	5

a Correlation is significant at the .01 level (2-tailed).

b Correlation is significant at the .05 level (2-tailed).

## 3 Results

The systematic literature search identified a total of 9 studies that met the inclusion criteria for the meta-analysis (Hajiabadi et al., 2023; J et al., 2019; Van Buyten et al., 2008; Kapural et al., 2015; Kriek et al., 2017; Reverberi et al., 2013; Jakobs et al., 2023; Jakobs et al., 2018; De Vloo et al., 2018). These studies were focused on the clinical outcomes and complications associated with r-IPGs used in neurostimulation surgeries.

The included studies varied in terms of their level of evidence, with 3 studies classified as case series (level 4 evidence) and 2 studies classified as randomized, double-blinded controlled trials (level 3 evidence). The studies encompassed a range of patients undergoing either SCS or DBS procedures using r-IPGs.

Data covering 5 studies with 288 patients for SCS, and 4 studies with 257 patients for DBS, and both the numerical values and proportional values of the complications seen in these patients on a per patient basis were calculated as a percentage, and the results are shown in Table 1.

**Fig. 2:**
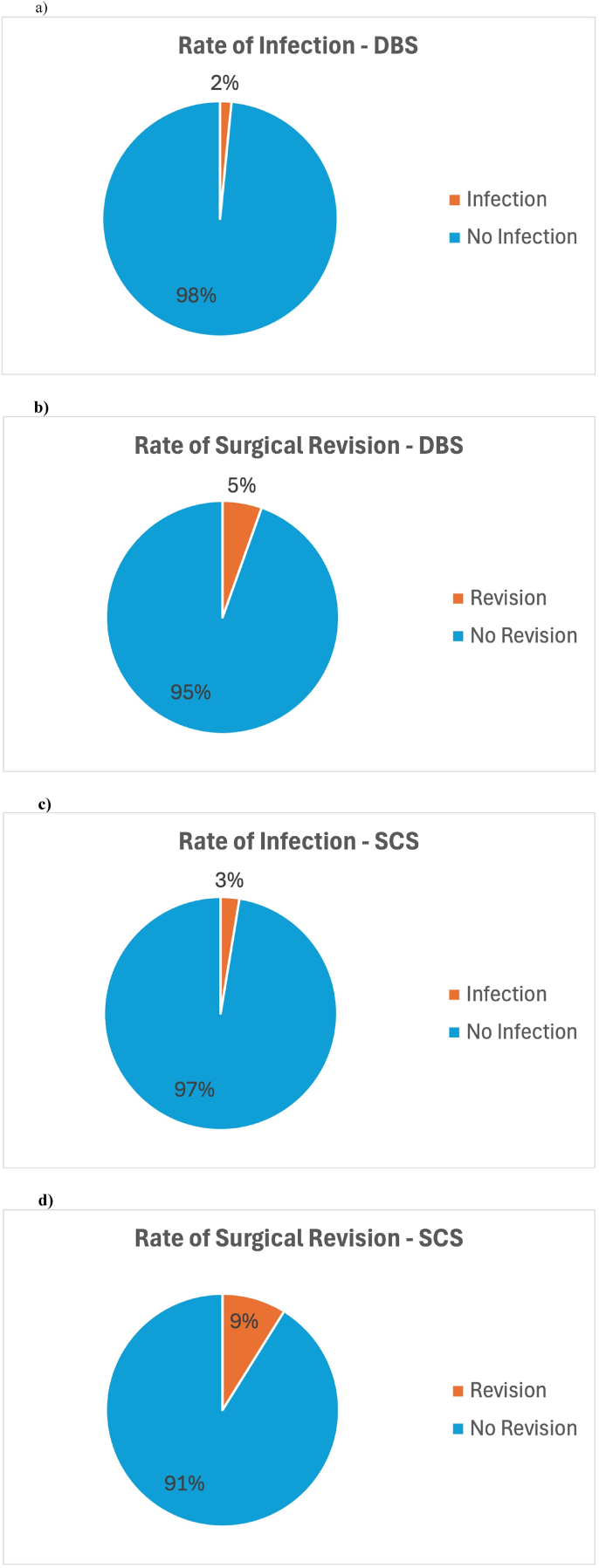
Surgery-related complications of rechargeable IPGs. Rates of infection and surgical revisions for both DBS (a & b) and SCS (c & d) therapy.

**Fig. 3:**
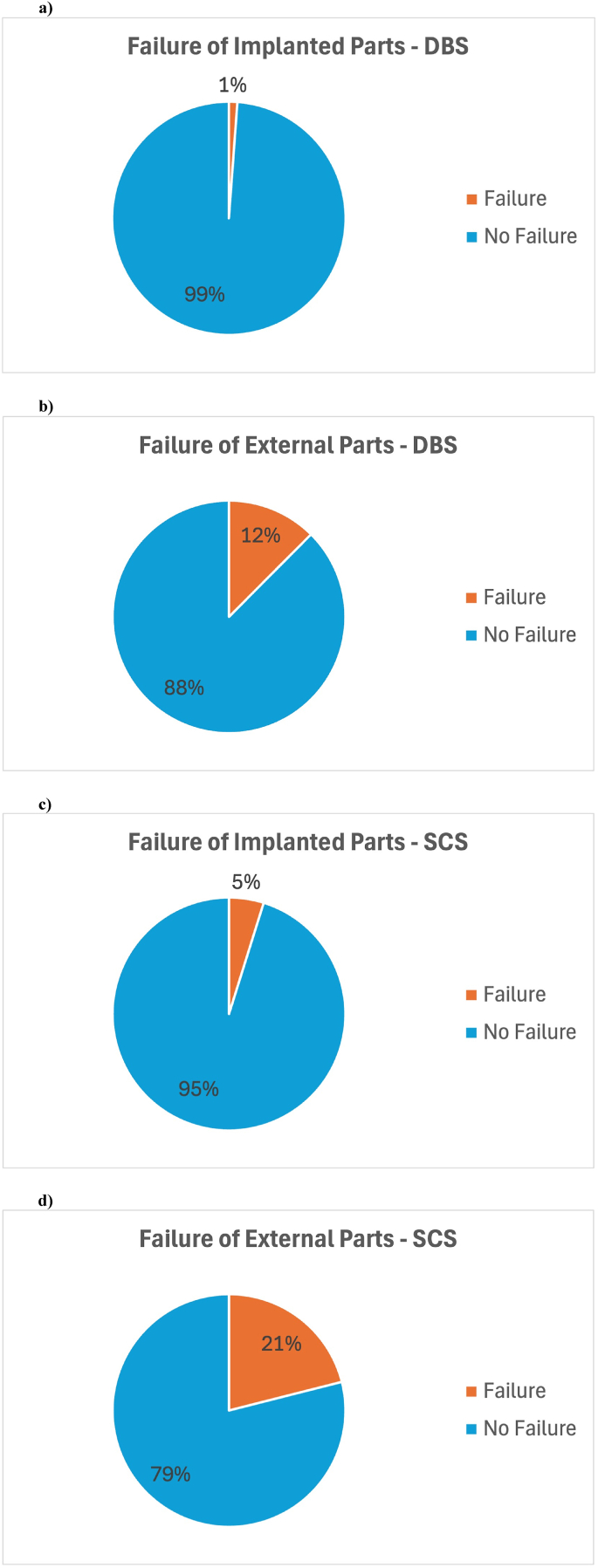
Hardware related complication for rechargeable IPGs. Rates of failure of implanted and explanted parts for both DBS (a & b) and SCS (c & d) therapy.

Descriptive statistics for SCS stimulation type and descriptive statistics for DBS stimulation type were given in Table 2, Table 3. Database analysis identified 9 studies with 288 SCS and 254 DBS patients that met the inclusion criteria. The average recharge time was 66.05 min for SCS and 126.5 min for DBS. The average interval between recharges was 4.75 days for SCS and 9.48 days for DBS. For SCS, the rates were: infection 2.6%, surgical revisions 8.87% (Fig. 2), implanted hardware failure 4.86%, external hardware failure 21.05% (Fig. 3), recharge interruptions 13.48%, and unintended interruptions 12.25%. For DBS, the rates were: infection 1.56%, surgical revisions 5.45% (Fig. 2), implanted hardware failure 1.17%, external hardware failure 12.45% (Fig. 3), recharge interruptions 10.51%, unintended interruptions 8.5%, and failed recharge attempts 3.89%.

While the average weekly battery charge burden (De Vloo et al., 2018) for DBS and SCS patients were quite similar [66.05 min∗(7/4.75 days) ≈ 97.34 min/week for SCS and 126.5 min∗(7/9.48 days) ≈ 93.41 min/week for DBS], the unweighted averages reveal that SCS battery charging duration was nearly half that of DBS (66 min vs. 126 min). However, the reverse was observed in the charging intervals, with DBS having a longer battery life between charges (9.48 days vs. 4.75 days).

Pearson correlation analyses were conducted to assess the relationships between different variables within the SCS and DBS stimulation types. The analyses revealed significant correlations between battery charging time and complications such as unintentional interruptions, infection rates, surgical revisions, hardware failures, interruptions due to recharging problems, and unsuccessful trials of recharging. Additionally, correlations were observed between surgical revisions and infection rates, hardware failures of external elements, interruptions due to recharging problems, and unintentional interruptions. These findings highlight the interconnectedness of various factors in influencing the outcomes and complications associated with r-IPGs in neurostimulation surgeries.

Based on the patient number and the calculated data for SCS and DBS accordance to Table 1, Pearson correlation results for SCS stimulation type and for DBS stimulation type were given in Table 4, Table 5.

The statistical analysis provided quantitative support for the observed associations and trends, reinforcing the significance of factors such as battery charging time, complications, and patient outcomes in the context of r-IPGs and neurostimulation procedures.

Correlation Analyses Results for SCS Stimulation Type.

• As battery recharge time increases, surgical revisions, interruptions because of recharging problems and unintentional interruptions increase and there is a perfect positive relationship between battery recharge time and them with 1.00.
• It was observed that as the interval between charges increases, surgical revisions increase (1.00), recharging problems and unintentional interruptions decreased (−1.00).
• It has been observed that as infections increase, hardware failures of implanted parts and interruption because of recharging in both instances infections with 1.00.
• It has been observed that as surgical revisions increase with interruption because of recharging problems, and there is an excellent positive relationship with 1.00.
• It has been observed that as hardware failures of external parts increases, interruption because of recharging problems increases and there is a perfect positive relationship between them with 1.00.
• As interruptions because of recharging problems increase, unintentional interruptions increase and there is a nearly perfect relationship between them with .997.

Correlation Analyses Results for DBS Stimulation Type.

• It has been observed that as infections increase, hardware failures of external parts, interruptions because of recharging problems and unsuccessful trials of recharging increase and there is positive correlation between them at a near perfect level of .923, .923 and .907 respectively.
• It has been observed that as surgical revisions increase, interruption because of recharging problems increase and there is positive correlation between them at a near perfect level of .983.

Overall, the results of the meta-analysis and review underscore the importance of r-IPGs in neurostimulation therapies, highlighting their potential benefits in mitigating complications, reducing re-operation rates, and improving patient outcomes compared to nr-IPGs (Constantoyannis et al., 2005; Videnovic and Metman, 2008; Baizabal Carvallo et al., 2012). However, careful monitoring of battery charge levels and adherence to charging procedures remain crucial to ensure optimal performance and minimize risks associated with r-IPGs.

## 4 Discussion

This meta-analysis and review evaluated the clinical outcomes and complications of r-IPGs used for neuromodulation. The research highlights the importance of this technology for their potential to reduce re-operation rates, lower disease-related risks, and improve patient outcomes.

It has been demonstrated that the long-term presence of an active implant within the skin and the subcutaneous tissue does not pose an infection risk (Goudman et al., 2014). Additionally, the infection rate increases with the number of IPG-replacement surgeries, with the rate reaching up to 20% for three or more replacements (Boviatsis et al., 2010; Thrane et al., 2014). Our results show that r-IPGs are associated a low complication need and especially a low rate of infections (the infection rate for SCS was 2.6%, with surgical revisions at 8.87%, while for DBS, the infection rate was 1.56%, with surgical revisions at 5.45%) (Helmers et al., 2018a). The benefit of r-IPGs however plays out in the long-term by which they can reduce the need for reoperation or infection-related interruption of therapy.

While the rates for surgical revisions and infections remained similar between SCS and DBS patients our study found that SCS patients recharged for much shorter but also more often compared to DBS patients. Calculating the charge burden for SCS and DBS patients from our results reveals a very comparable charge burden (97.34 min/week vs. 93.41 min/week). This finding is unexpected as SCS and DBS usually require different stimulation parameters and implantation sites for IPGs and while DBS usually is a continuous treatment, SCS patients elect to switch stimulation off more regularly. Why SCS patients on average recharge for shorter more often compared to DBS patients cannot be answered from our analysis.

Longer battery charging times as well as shorter intervals between recharges were associated with recharging problems and unintentional interruptions of therapy for SCS. Therefore, charging strategies that include recharging at intermediate battery charge levels may be favourable.

To ensure the effectiveness of r-IPGs, battery charge levels must be checked regularly, and charging procedures must be followed. Otherwise, unexpected situations such as battery problems may occur, resulting in treatment interruptions. For both SCS and DBS unintentional interruptions of stimulation due to problems with recharging affected every 10th patient. Different factors such as cognitive impairments in PD patients or opioid-based co-medication in chronic pain patients may be contributing to this rate.

While there is data from this meta-analysis on the rate of unintentional interruptions of therapy with r-IPGs due to battery depletion, there is no data in the literature for the corresponding event with nr-IPGs: patients presenting for IPG-replacement surgeries due to complete depletion of the battery.

Therefore, it is still unknown whether r-IPGs or nr-IPGs are overall more prone to interruptions of therapy. Patients with r-IPGs however have for the most part the option to restart stimulation by performing a charge of the neurostimulator while patients with nr-IPGs always require replacement surgery and help from surgeons and health-care professionals.

Another issue we observed in our study is the significant impact of the devices used in DBS and SCS interventions on complications and the patients' benefit from these interventions. As shown in our study, internal and external parts need to be considered and optimized separately, as the external parts of the r-IPG system (charger, charging belt, patient programmer) fail much more often than the implantable parts (IPG, leads, extensions) and failure of external parts was directly correlated with unintentional interruptions of therapy. Manufacturers should therefore focus on improvements of the external parts of the charging system as these were most often the reason for interruptions of therapy.

Our meta-analysis has several limitations. The number of publications that investigate aspects of r-IPGs as the main point of interest is low hence the comparatively small number of investigated patients. Furthermore, there is not a single study that compares nr-IPGs an r-IPGs with regards to complications and patient satisfaction head-to-head in a prospective manner in a homogenous patient population. This might help to find factors that favour one type of stimulator over the other.

The results from this systematic review may inform future studies to find meaningful endpoints and aid in the calculation for relevant study group sizes.

Until such data exist national or international guidelines and recommendations based on the current literature and expert opinion might be developed that guide physicians and health care professionals in the decision-making process regarding IPG choice.

In these guidelines, pre-op and post-op processes should be laid out, and patient education and follow-up should be standardized.

This optimization should aim to increase the patient's comfort, especially post-operative complications, and minimize the financial burden.

Discussions on these topics may help us better understand the role of r-IPGs in neuromodulation therapies and enhance the clinical applications of this technology.

## Statement of ethics

An ethics statement is not applicable because this study is based exclusively on published literature.

## Author contributions

Denizhan Özgan wrote the first draft of the manuscript and performed the literature review, data extraction and statistical analysis. Sandro Krieg supervised the entire project and performed proof-reading of the final draft. Martin Jakobs conceptualized the project performed reviews and corrections for first draft of the manuscript.

## Data availability statement

The data that support the findings of this study are not publicly available due them containing information that could compromise the privacy of research participants but are available from the corresponding author (MJ) upon reasonable request.

## Funding sources

Funding for this systematic review was provided by the Department of Neurosurgery of the University Hospital Heidelberg. The funder had no role in the design, data collection, data analysis, and reporting of this study.

## Declaration of competing interest

The authors declare the following financial interests/personal relationships which may be considered as potential competing interests: Martin Jakobs has received educational grants from Boston Scientific and inomed Medizintechnik as well as speaking grants from Precisis GmbH. If there are other authors, they declare that they have no known competing financial interests or personal relationships that could have appeared to influence the work reported in this paper.
